# Taxonomy of Atlantic Central African orchids 2. A second species of the rare genus *Distylodon* (Orchidaceae, Angraecinae) collected in Cameroon

**DOI:** 10.3897/phytokeys.36.7225

**Published:** 2014-04-09

**Authors:** Vincent Droissart, Phillip J. Cribb, Murielle Simo-Droissart, Tariq Stévart

**Affiliations:** 1 Institut de Recherche pour le Développement (IRD), Unité Mixte de Recherche AMAP (Botanique et Bioinformatique de l’Architecture des Plantes), Boulevard de la Lironde, TA A-51/PS2, F-34398 Montpellier Cedex 5, France; 2 Missouri Botanical Garden, Africa & Madagascar Department, P. O. Box 299, St. Louis, Missouri 63166-0299, U.S.A.; 3 Plant Systematic and Ecology Laboratory, Higher Teacher’s Training College, University of Yaoundé I, Yaoundé, Cameroon; 4 Herbarium et Bibliothèque de Botanique africaine, CP 265, Université Libre de Bruxelles, Boulevard du Triomphe, B-1050, Brussels, Belgium; 5 Herbarium, Royal Botanic Gardens, Kew, Richmond, Surrey, TW9 3AB, UK; 6 Botanic Garden, Meise, Domein van Bouchout, Nieuwelaan 38, B-1860 Meise, Belgium

**Keywords:** Angraecoid, Cameroon, Campo-Ma’an National Park, IUCN Red List Categories and Criteria, new species

## Abstract

While conducting field inventories in South Cameroon, we collected two specimens of a new species that we considered to belong to the genus *Angraecopsis*. Afterwards, a careful examination of specimens housed at main herbaria, along with the nomenclatural types, allows us to place it in *Distylodon*, a monotypic genus previously known from East Africa. *Distylodon sonkeanum* Droissart, Stévart & P.J.Cribb, **sp. nov.** was collected in the lowland coastal forest of Atlantic Central Africa. It is known from a single locality in the surroundings of the Campo-Ma’an National Park. The species differs from *D. comptum*, by its several-flowered inflorescences, longer leaves and spur, and shorter pedicel and ovary. The species appears to be rare and is assessed as Critically Endangered [CR B2ab(iii)] according to IUCN Red List Categories and Criteria. New field investigations are required to attempt to find it in the low-elevation parts of the Campo-Ma’an National Park in Cameroon.

## Introduction

During fieldwork conducted in April 2007 by the first and the third authors (Droissart 2009) in the surroundings of the Campo Ma’an National Park, near the village of Bifa (South Region, Cameroon), two living specimens of an unknown orchid were collected without flowers and were thus cultivated in an orchid shadehouse at Yaoundé. These specimens flowered in July 2007 and were initially placed in *Angraecopsis* through the trilobate shape of the lip. Then, a detailed examination and comparison with material at the Orchid Herbarium of the Royal Botanic Gardens, Kew, and with literature (e.g. Summerhayes 1933, 1951, Szlachetko and Olszewski 2001), allowed us to identify these specimens as belonging to a new species of the monotypic genus *Distylodon* Summerh.

Complementary surveys in the same area and examination of closely related material preserved at BR, BRLU, K, P, WAG and YA (herbaria acronyms according to Thiers continuously updated) did not reveal any additional specimens of this novelty. Seven years after our discovery in Cameroon, we describe the new species collected from a single locality, resulting in the present paper. This paper represents the second in a series of publications based on recent intensive field work (Stévart 2003, Droissart 2009) and focusing on collections-based taxonomic revisions of Orchidaceae in Atlantic Central Africa.

## Materials and methods

Collections of BR, BRLU, K, P, WAG and YA were examined. Measurements, colors and other details given in the description are based on living material, alcohol-preserved specimens, and data derived from field notes. Morphological investigation used a Nikon SMZ645 stereomicroscope. The conservation status of the species was assessed by applying the IUCN Red List Categories and Criteria (IUCN 2001, 2008).

## Taxonomic treatment

### Distylodon sonkeanum

Droissart, Stévart & P.J.Cribb sp. nov.

urn:lsid:ipni.org:names:77137691-1

http://species-id.net/wiki/Distylodon_sonkeanum

Figs 1 , 2

#### Diagnosis.

*Distylodon sonkeanum* Droissart, Stévart & P.J.Cribb, is close to *Distylodon comptum* Summerh. but differs from it in having several-flowered inflorescences, longer leaves and spur, and shorter pedicel and ovary.

#### Type.

Cameroon. Bifa (piste sur la route Kribi-Ebolowa), à 5 km au SE de Zingui, le long d’une piste de chasseurs entre les rivières Nyété et Nyamefoo, 02°41.308'N, 010°16.406'E, 5 July 2007, *Droissart, Stévart & Simo M. 585* (holotype: BRLU!; isotype: YA!).

#### Description.

Dwarf epiphytic herb. Stem short, up to 8 mm long, 2–2.5 mm in diameter, leafy, unbranched. Roots more than 120 mm long, 1–1.8 mm in diameter, numerous, distributed at the base of the plant, unbranched. Leaves few (up to 5), 25–37 × 6–11 mm, obliquely narrowly elliptic, slightly coriaceous, with entire margins, with an indistinct and irregular reticulate venation; leaf apex obliquely subacute, unequally bilobed, with distance between the two lobes less than 1 mm. Inflorescences up to 41 mm long, 2- to 5-flowered, almost horizontal to pendant, unbranched, emerging at the base of the stem, with peduncle 13 mm long; floral internode about 6–7 mm, one flower per node; rachis terete. Bracts up to 1.2 mm long, tubular. Flowers 17–25 × 6–8 mm, not opening widely, green, the larger one situated at the apex of the inflorescence. Ovary and pedicel not twisted, 5–7 mm long. Dorsal sepal 4–7 × 1.6–1.9 mm, linear lanceolate, acute, slightly to markedly reflexed, with entire margins. Lateral sepals 5.5–9 × 1.8–2.0 mm, obliquely linear-lanceolate, acute, slightly to markedly reflexed, with entire margins. Petals 3.0–4.8 × 1.1–1.3 mm, linear lanceolate, acuminate, slightly curved forward, with entire margins. Lip 4.5–6.0 × 2.0–3.5 mm, slightly to markedly trilobed in the basal half; side lobe obliquely triangular, subacute to acute, 0.8–1.8 mm long; mid lobe much longer, 3–4 mm long, linear-subulate, acuminate, fleshy, somewhat curved forward; spur 13.5–18.0 mm long, 0.8–1.1 mm in diameter, cylindrical, straight, slightly inflated in the apical part in larger flowers, apex rounded. Column 1–1.2 × 1.0 mm, almost cylindrical with truncate apex. Rostellum 0.3 mm long, consists of two erect, subulate teeth or fangs. Anthercap 1.0 × 1.0 mm, deltoid. Pollinia two, spherical. Viscidia two, with two stipites 0.7–0.8 mm long, independent of each other, flattened, bifurcate.

**Figure 1.**
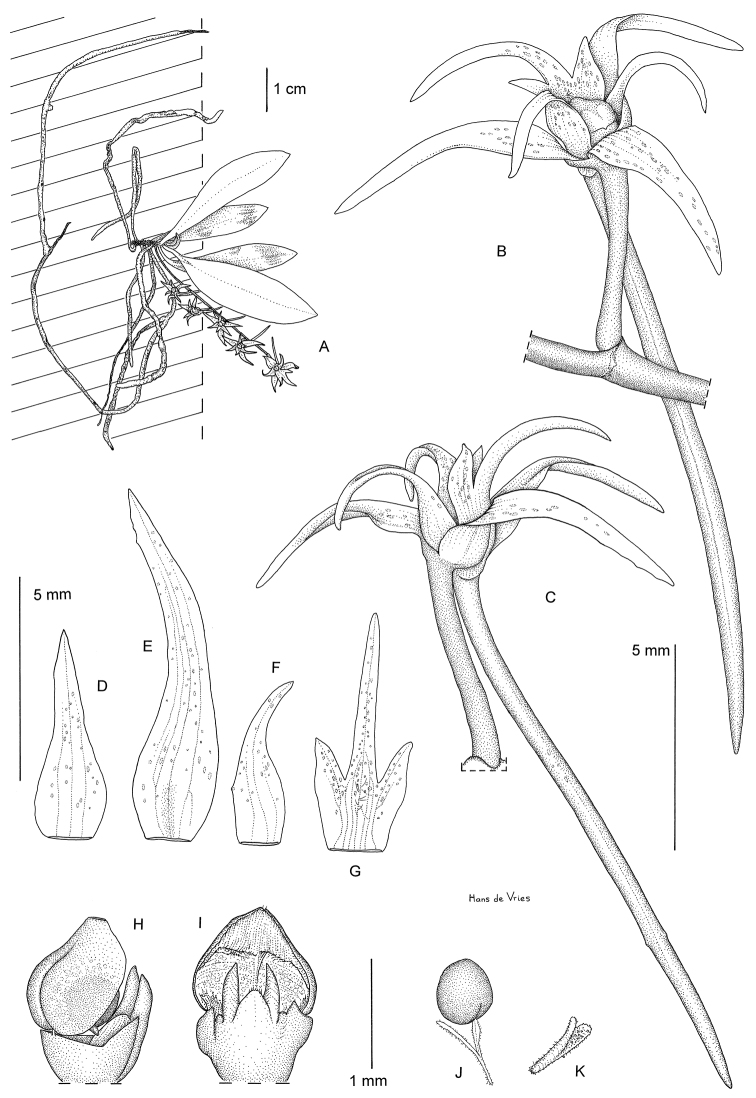
Illustration of *Distylodon sonkeanum* (*Droissart, Stévart & Simo M. 585*): **A** *habitus* **B** flower, diagonal view **C** flower, side view **D** dorsal sepal **E** lateral sepal **F** petal **G** lip **H** column with pollinium, sideview **I** column with anthercap, frontal **J** pollinium with stipe **K** stipe.

**Figure 2.**
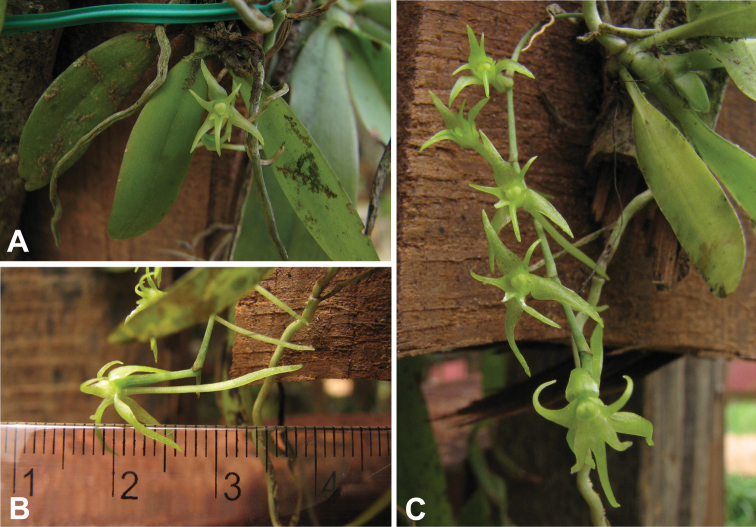
Photographs of living specimen of *Distylodon sonkeanum*: **A** front view of the flower **B** side view of the flower **C** inflorescence.

#### Distribution and habitat.

**Figure 3.**
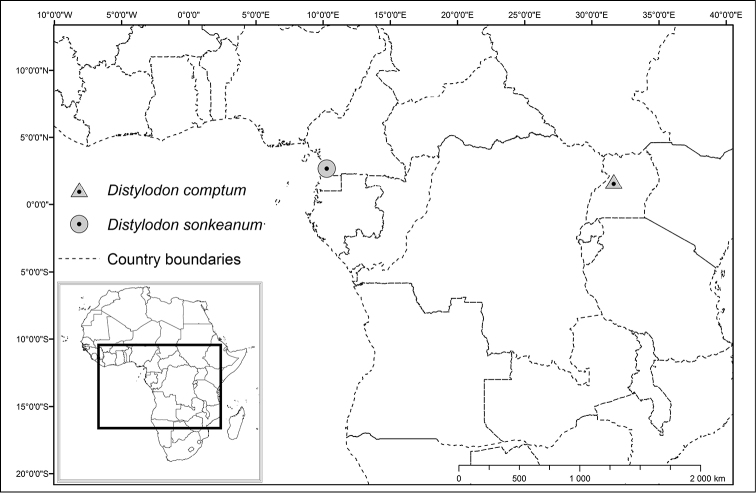
Distribution of *Distylodon sonkeanum* and *Distylodon comptum* in tropical Africa.

*Distylodon sonkeanum* is endemic to the coastal part of the Lower Guinea Domain (White 1979) of the Guineo-Congolian Region. It is known from a single locality in South Region of Cameroon (Fig. 3). The only population known so far was found in the lowland evergreen forest at 100 m elevation, growing epiphytically on a fallen branch.

#### Conservation.

IUCN red list category: Critically Endangered, [CR B2ab(iii)]. *Distylodon sonkeanum* is only known from one very restricted subpopulation in Cameroon which represents one ‘location’. The main threats known to the species in the southern Cameroon are deforestation for logging and oil palm (*Elaeis guineensis* Jacq.) and rubber (*Hevea brasiliensis* (Willd. Ex A. Juss.) Müll.Arg.) plantations, resulting from a strong international demand. The ongoing loss of the forest leads us to expect a continuous decline of its habitat in the only known locality, and therefore of its extent of occurrence and area of occupancy. Moreover, this location is accessible to local residents and through their practices of shifting agriculture and small-scale timber exploitation; they are gradually transforming this area into secondary forest.

*Distylodon sonkeanum* appears to be rare but, due to its inconspicuous habit, further fieldwork is required to ascertain its conservation status more objectively. Considering the general habitat of *Distylodon sonkeanum*, it is to be hoped that more specimens and additional sites will be found in the low-elevation parts of the adjacent Campo Ma’an National Park. Based on our current knowledge of the ecology and the distribution of the species (one location and AOO less than 10 km^2^), and using the Criteria B of the IUCN Red List Categories and Criteria, the species was assessed as CR B2ab(iii).

#### Etymology.

The species epithet ‘*sonkeanum*’ refers to the Professor Bonaventure Sonké of the University of Yaoundé I, who is an internationally recognized taxonomist, specialized in the Rubiaceae’s family. He has collected extensively in the Bipindi-Akom II area, and adjacent Ngovayang massif and Campo-Ma’an National Park. He was one of the first to highlight the high biodiversity of this area and he greatly facilitated our work in Cameroon.

### Key to the species of *Distylodon*

**Table:** 

1	Inflorescence several-flowered (2- to 5-flowered), leaves 25–37 mm long, spur 13.5–18.0 mm long, pedicel and ovary 5–7 mm long	*Distylodon sonkeanum*
2	Inflorescences one-flowered, leaves 7–17 mm long, spur 6.5 mm long, pedicel and ovary 9 mm long	*Distylodon comptum*

## Discussion

The genus *Distylodon* was established about 50 years ago by Summerhayes (1966). Before our discovery, it was represented only by *Distylodon comptum* Summerh., known from one specimen collected in Uganda in 1944 (*Eggeling 5381*, holotype K!). As suggested by its etymology, this genus is mainly characterized by the shape of the rostellum which is bilobated, the two lobes standing up parallel to one another forming two narrow almost subulate acute teeth or fangs (Fig. 1H, I).

*Distylodon sonkeanum* is easily distinguished from the only taxon described in the genus so far, *Distylodon comptum*. The new species produces pluriflorous inflorescences, longer leaves and spur, and shorter pedicel and ovary. As pointed out by Summerhayes (1966), the genus appears to be closely allied to *Angraecopsis* Kraenzl. Both genera present short stemmed plants with distichous, conduplicate leaves with unequally lobed tips; their inflorescences are short bearing small, spurred and pale yellow to green colored flowers. Nevertheless, the genus *Distylodon* can be distinguished by its sepals with similar shape and size, and the characteristic shape of its rostellum. Further molecular evidences are required to test the monophyly of *Distylodon*. Unfortunately, no material suitable for DNA studies has been collected so far and consequently the phylogenetic placement of the species remains to determine.

The distance between the localities of *Distylodon sonkeanum* and *Distylodon comptum* is more than 2,000 km (Fig. 3). The Albertine Rift is well known for the concentration of many narrow endemics, being a hotspot of plant diversity in East Africa (Brooks et al. 2004). The territories surrounding the Campo-Ma’an National Park also harbor many orchids that are endemic to Atlantic Central Africa (Droissart 2009), and our discovery stresses the need of further explorations and plant protection in this area. The large gap between the two taxa, covering the Congolian sub-centre of endemism (White 1979), remains largely unsampled and future botanical explorations may reveal that the geographic disjunction between the two species is not as large as we may believe today.

## Supplementary Material

XML Treatment for Distylodon sonkeanum
